# Prevention of cytomegalovirus infection after solid organ transplantation: a Bayesian network analysis

**DOI:** 10.1186/s12941-020-00372-0

**Published:** 2020-08-05

**Authors:** Yu Zhang, Tao Zhou, Mingzhu Huang, Guangxiang Gu, Qiang Xia

**Affiliations:** Department of Liver Surgery and Liver Transplantation Center, Ren Ji Hospital, School of Medicine, Shanghai Jiao Tong University, 1630 Dongfang Road, Pudong New District, Shanghai, 200 127 China

**Keywords:** Cytomegalovirus, Solid organ transplantation, Bayesian network analysis, Prevention

## Abstract

**Background:**

Cytomegalovirus infection is one of the most common complications after solid organ transplantation. There have been several classes of antiviral drugs for the prevention of cytomegalovirus infection, such as acyclovir, valacyclovir, ganciclovir and valganciclovir.

**Methods:**

We searched relevant prospective and multi-armed studies on PubMed from Jan. 1984 up to Mar. 2018.

**Results:**

Seventeen prospective studies involving 2062 patients were included in the analysis. In the case of cytomegalovirus infection, the ganciclovir group (OR = 0.24, 95% CI 0.09–0.57) and the valacyclovir group (OR = 0.20, 95% CI 0.04–0.69) provided significantly better outcomes than the control group. The ganciclovir (OR = 0.37, 95% CI 0.13–0.86) and valacyclovir groups (OR = 0.31, 95% CI 0.07–0.98) showed moderate superiority compared to the acyclovir group. As for cytomegalovirus disease, the ganciclovir, valacyclovir and valganciclovir groups showed significant advantages compared with the control group (ganciclovir group: OR = 0.17, 95% CI 0.07–0.31, valacyclovir group: OR = 0.08, 95% CI 0.01–0.33, valganciclovir group: OR = 0.14, 95% CI 0.02–0.45). Similarly, the ganciclovir group (OR = 0.38, 95% CI 0.12–0.71) and the valacyclovir group (OR = 0.17, 95% CI 0.03–0.72) showed better results than the acyclovir group.

**Conclusion:**

Valacyclovir showed to be the most efficient antiviral for the prevention of cytomegalovirus infection and disease. Additional studies are required to evaluate putative side effects associated with valacyclovir administration.

## Background

Disease caused by cytomegalovirus (CMV) is one of the most common complications after solid organ transplantation [1]. Initially, the infection is mainly asymptomatic, but can progress to disease which is characterized by symptomatic viremia or tissue-invasive disease [2]. CMV mostly affects the lungs, liver, and digestive system, followed by a series of secondary side effects [3]. CMV can also indirectly cause some other adverse outcomes, such as allograft rejection and opportunistic infections, which eventually lead to reduced allograft survival and increased mortality [1]. Thanks to the improvement in the diagnostic techniques, CMV infection can be detected earlier [4, 5]. The risk of CMV infection varies widely, depending on the serological status of the donors(D) and the recipients(R): (D+/R-high risk, D−/R+ and D+/R+ intermediate risk, D−/R-low risk) [6]. Currently, there are two main strategies for CMV infection prevention after solid organ transplantation, namely, universal prophylaxis and preemptive therapy. Universal prophylaxis involves the administration of antiviral agents to all patients or to a selected cohort of patients at high risk of CMV infection after solid organ transplantation. Preemptive therapy is administered to recipients starting with CMV viremia with or without symptoms of CMV infection after solid organ transplantation [7]. Although ganciclovir and valganciclovir are recommended for CMV treatment according to the third international consensus guidelines on the management of cytomegalovirus in solid organ transplantation, several antiviral agents are currently in use for CMV prevention and treatment, including acyclovir, valacyclovir, ganciclovir and valganciclovir. However, these antiviral drugs are associated with toxicity and severe side effects, such as leukopenia, thrombocytopenia, renal dysfunction, neuropsychiatric symptoms, and drug resistance [8]. Nowadays, there are no guidelines for standard therapy for the prevention of CMV infection after solid organ transplantation due to the lack of prospective head-to-head studies comparing the effectiveness of some of antiviral drugs. In an attempt to understand more about the problem, we performed a Bayesian network meta-analysis to draw an indirect comparison of evidence and provide some clues for the prevention of CMV infection after solid organ transplantation.

## Methods

### Search strategy

Two different investigators independently searched PubMed (1984.1–2018.3). Our search is based on ((“Organ Transplantation” [Mesh]) AND “Cytomegalovirus” [Mesh]) AND “Acyclovir” [Mesh], ((“Organ Transplantation” [Mesh]) AND “Cytomegalovirus” [Mesh]) AND “Ganciclovir” [Mesh], ((“Organ Transplantation” [Mesh]) AND “Cytomegalovirus” [Mesh]) AND “valacyclovir” and ((“Organ Transplantation” [Mesh]) AND “Cytomegalovirus” [Mesh]) AND “valganciclovir”. We went through each title and abstract carefully to collect the articles, so that they could be analyzed systematically and comprehensively. In the cases where this could not be done based on the title and abstract of the articles, we analyzed the full text in order to maximize the accuracy of the selection.

### Selection criteria

Two different investigators independently collected studies that meet our requirements. When there was a disagreement, we turned to a third party for solutions. We chose prospective multi-arm studies with full text in English, and excluded the studies comparing different doses and maintenance times of the same antiviral drugs. Besides this, we also excluded the studies performing interventions with multiple antiviral drugs in the same arm.

The baseline characteristics of the study consisted of the following: study name, journal, first author, year, transplanted organ, transplantation center, prevention strategies, antiviral drug, dose, intervention time, number of samples, follow-up time, and immunosuppressive regimen. The prevention strategies included prophylaxis therapy and preemptive therapy. Prophylactic therapy refers to a preventive measure after solid organ transplantation, regardless of whether the patients were seropositive for CMV. Preemptive therapy was for recipients presenting CMV viremia with or without symptoms of CMV infection after solid organ transplantation. Deferred therapy means antiviral measures after the onset of CMV disease, which is similar to placebo therapy. So, we defined placebo and deferred therapy as control group.

There were defined two primary endpoints, CMV infection and CMV disease. The former is the presence of the virus replication which is defined as virus isolation or detection of viral proteins (antigens) or nucleic acid in any body fluid or tissue specimen by qualitative or quantitative PCR, while the latter is accompanied by clinical manifestations. CMV disease is categorized into two types, the first consist in a viral syndrome (fever, malaise, leukopenia, and/or thrombocytopenia) and the second type, a tissue invasive disease. We also focused on acute rejection, graft dysfunction, mortality and the side effects of the antiviral drugs (leukopenia, thrombocytopenia, neuropsychiatric symptoms, renal function).

### Statistical analysis

First, we conducted a direct pairwise comparison with random effects models. The heterogeneity was assessed by I^2^ statistic (low-degree: 25–49%; moderate-degree: 50–75%; high-degree: > 75%) [9]. All studied endpoints were dichotomous variables. Odds ratio (OR) with 95% confidential interval (95% CI) were applied a measure of effect size. We used a funnel plot asymmetry to measure publication bias.

Second, the Bayesian network meta-analysis was performed with GeMTC in a random-effect or fixed-effects model using Markov chain Monte Carlo methods. For each outcome, four Markov chains with different starting values were run in parallel for 50,000 iterations to obtain the posterior distribution. We used 20,000 burn-ins and a thinning interval of 10 for each chain. To verify if there is inconsistency, a loop inconsistency-specific approach was used to evaluate the difference between direct and indirect estimates for specific comparisons [10]. A relative Odds Ratio (ROR) close to 1, indicate that the direct comparison and indirect comparison results are consistent. Otherwise, we retrospectively analyzed the sources of inconsistency. The direct and indirect results were compared to roughly assess the consistency between the direct and indirect evidence, which was statistically confirmed by node-splitting analyses. Node-splitting analysis was used because it is more sophisticated and robust in complex networks with multi-arm trials. Finally, a comparison-adjusted funnel plot was used to assess publication bias. Statistical analyses were performed using STATA (version 13.0), Review Manager (version 5.3) and GeMTC (version 0.14.3). All tests were two-sided. Results were considered to have statistically significant when p-values were < 0.05 or 95% CIs excluding one.

## Results

### Eligible studies and patient characteristics

**Fig. 1:**
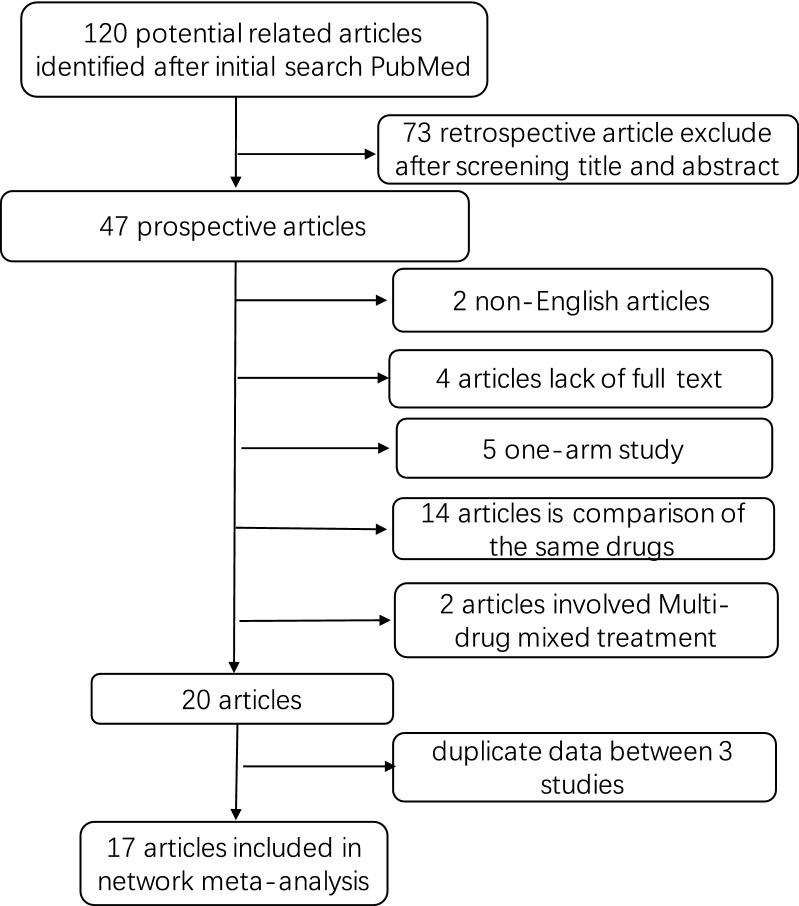
Flow chart of study selection. We searched prospective and multi-armed studies on PubMed from Jan 1984 up to Mar. 2018. 17 prospective studies involving 2062 patients were included in our network meta-analysis

**Fig. 2:**
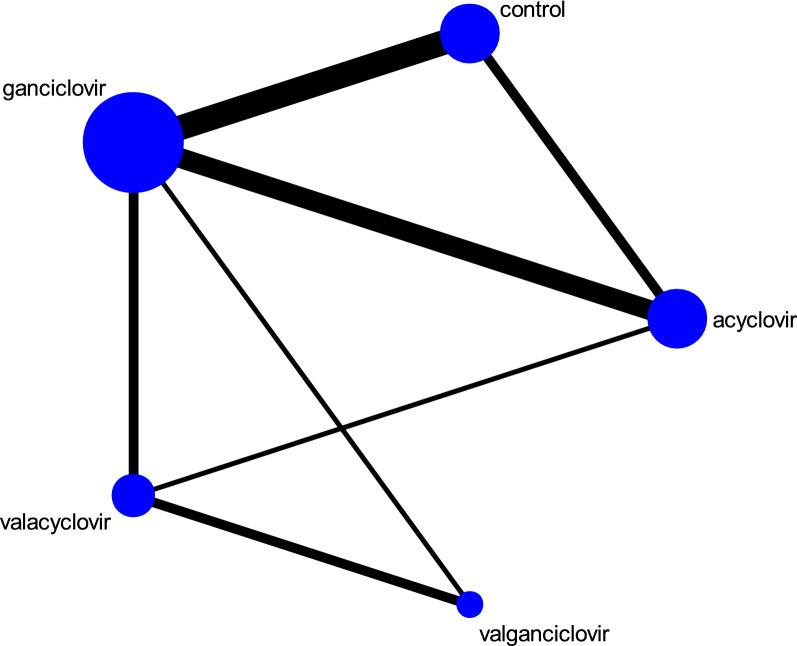
Network of direct pairwise comparisons between different antiviral drugs. Different nodes represent different prevention measures and the size of the nodes corresponds to the number of patients. The line represents a direct comparison between the two prevention measures and the thickness of the line is consistent with the number of direct comparisons of the two prevention measures

A total of 120 related studies were retrieved from PubMed database. Through carefully analysis, 17 studies were included in the meta-analysis. The detailed screening process is presented in Fig. 1. The 17 independent studies involving 2062 patients encompass different antiviral drugs. The characteristics of the 17 studies are described in Table 1. Two studies involved different organs, while the remaining 15 studies involve only one transplanted organ. Of these 17 randomized controlled trials, seven were randomized controlled trials and all were two-armed. The network diagram of the comparison between the antiviral drugs is shown in Fig. 2. There are seven subgroups for direct comparisons between the antiviral drugs. The specific network diagram of the different outcomes in depicted in Additional file 1: Figure S1.

**Table 1:** Characteristics of the included studies

First author	Organ		Prevention strategy	Antiviral drug	Dose (mg/days)	Intervention time (days)	Number of samples	D−/R+ (%)	Follow-up time (days)	Immunosuppressive regimen
Acyclovir vs control										
Balfour [6]	Renal		Prophylaxis	Acyclovir	3200	84	53		365	Azathioprine, cyclosporine, prednisone, antiymphoblast globulin
				Control	3200	84	51		365	Azathioprine, cyclosporine, prednisone, antiymphoblast globulin
Barkholt [7]	Liver		Prophylaxis	Acyclovir	3200	84	28	2 (7)	168	Cyclosporine, azathioprine, steroids
				Control	3200	84	27	3 (11)	168	Cyclosporine, azathioprine, steroids
Acyclovir vs ganciclovir										
Singh [8]	Liver		Prophylaxis	Acyclovir	3200	7	24	7 (30)	168	Tacrolimus, methylprednisolone
			Preemptive	Ganciclovir	10 mg/kg	168	23	8 (33)	168	Tacrolimus, methylprednisolone
Duncan [9]	Lung		Prophylaxis	Acyclovir	3200	90	12		742 ± 44	Azathioprine, cyclosporine, antilymphoblast globulin/antilymphocyt globulin, methylprednisolone
			Prophylaxis	Ganciclovir	5 mg/kg 5 days/week	90	13		690 ± 85	Azathioprine, cyclosporine, antilymphoblast globulin/antilymphocyt globulin, methylprednisolone
Rubin [10]	Renal, heart, liver		Prophylaxis	Acyclovir	1200	84	78	77 (100)	365	
			Prophylaxis	Ganciclovir	3000	84	77	78 (100)	365	
Winston [11]	Liver		Prophylaxis	Acyclovir	3200	100	109		365	Tacrolimus/cyclosporine, corticosteroids, azathioprine
			Prophylaxis	Ganciclovir	3000	100	110		365	Tacrolimus/cyclosporine, corticosteroids, azathioprine
Acyclovir vs valacyclovir										
Egan [12]	Heart		Prophylaxis	Acyclovir	800	90	13	1 (7)	180	Azathioprine, cyclosporine, prednisolone
			Prophylaxis	Valacyclovir	2000	90	14	0 (0)	180	Azathioprine, cyclosporine, prednisolone
Ganciclovir vs control										
Merigan [13]	Heart		Prophylaxis	Ganciclovir	10 mg/k + 6 mg/kg/days 5 days/week	14 + 14	76	19 (25)	120	Corticosteroids, azathioprine, cyclosporine, OKT3 antibody
			Prophylaxis	Control	10 mg/k + 6 mg/kg/days 5 days/week	14 + 14	73	16 (22)	120	Corticosteroids, azathioprine, cyclosporine, OKT3 antibody
Hibberd [14]	Renal		Preemptive	Ganciclovir	2.5 mg/kg/days	9	64		180	Azathioprine, cyclosporine, prednisone, methylprednisolone
			Preemptive	Control	0	0	49		180	Azathioprine, cyclosporine, prednisone, methylprednisolone
Gane [15]	Liver		Prophylaxis	Ganciclovir	3000	98	150	21 (14)	180	Cyclosporine/tacrolimus
			Prophylaxis	Control	3000	98	154	25 (16)	180	Cyclosporine/tacrolimus
Paya [16]	Liver		Preemptive	Ganciclovir	3000	56	35	9 (26)	110	
			Preemptive	Control	3000	56	34	11 (32)	110	
Sagedal [17]	Renal		Preemptive	Ganciclovir	3000	49	42	5 (12)	365	Tacrolimus/cyclosporine, prednisolone
			Deferred	Control	3000	28	38	3 (8)	365	Tacrolimus/cyclosporine, prednisolone
Ganciclovir vs valacyclovir										
Pavlopoulou [18]	Renal		Prophylaxis	Ganciclovir	3000	90	40	6 (14)	180	Tacrolimus, MMF, methylprednisolone
			Prophylaxis	Valacyclovir	8000	90	43	6 (15)	180	Tacrolimus, MMF, methylprednisolone
Reischig [19]	Renal		Prophylaxis	Ganciclovir	3000	90	36	5 (14)	730	Tacrolimus, MMF, azathioprine
			Prophylaxis	Valacyclovir	8000	90	35	4 (11)	730	Tacrolimus, MMF, azathioprine
Ganciclovir vs valganciclovir										
Paya [20]	Heart, liver, renal, pancreas		Prophylaxis	Ganciclovir	3000	100	127	245 (100)	365	
			Prophylaxis	Valganciclovir	900	100	245	127 (100)	365	
Valganciclovir vs valacyclovir										
Reischig [21]	Renal		Prophylaxis	Valganciclovir	900	90	60	7 (11)	365	
			Prophylaxis	Valacyclovir	8000	90	59	4 (6)	365	
Reischig [22]	Renal		Preemptive	Valganciclovir	1800	14	36	6 (16)	1095	Tacrolimus/cyclosporine, MMF
			Prophylaxis	Valacyclovir	8000	90	34	4 (12)	1095	Tacrolimus/cyclosporine, MMF

*MMF* mycophenolate mofetil, *OKT3* muromonab-CD3

### Network meta-analysis between different intervention strategies

**Fig. 3:**
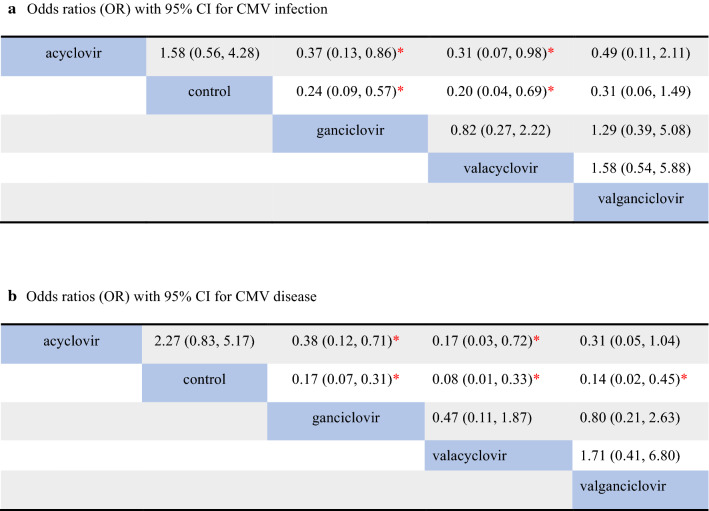
The results of the Bayesian network meta-analysis about CMV infection (**a**) and CMV disease (**b**). Each result is a comparison between the column-defining prevention measure and the row-defining the prevention measure. We highlight the data with significant statistical difference (p < 0.05) by *. *CI* confidence interval, *CMV* Cytomegalovirus. We should read result from right to left

The results of the network meta-analysis come from original studies. For CMV infection after solid organ transplantation, 14 studies were included in the analysis. Three studies were excluded since the outcomes of infection were not shown [11–13] (Fig. 3a). The ganciclovir group (OR = 0.24, 95% CI 0.09–0.57) and the valacyclovir group (OR = 0.20, 95% CI 0.04–0.69) performed significantly better than the control group, while the valganciclovir group (OR = 0.31, 95% CI 0.06–1.49) and the acyclovir group (OR = 0.63, 95% CI 0.23–1.78) show no significant advantage compared to the control group. Moreover, the ganciclovir (OR = 0.37, 95% CI 0.13–0.86) and valacyclovir groups (OR = 0.31, 95% CI 0.07–0.98) showed moderate superiority compared to the acyclovir group. However, the comparison between the valacyclovir group and the ganciclovir group did not show significant differences in their efficacy and safety (OR = 0.82 95% CI 0.27–2.22).

In respect to the occurrence of CMV disease after transplantation, all 17 research studies were included in the analysis. As we can observe in Fig. 3B, the ganciclovir, valacyclovir and valganciclovir groups showed significant advantages over the control group (ganciclovir group: OR = 0.17, 95% CI 0.07–0.31; valacyclovir group: OR = 0.08, 95% CI 0.01–0.33; valganciclovir group: OR = 0.14, 95% CI 0.02–0.45). There were still no significant differences between the acyclovir group and the control group (OR = 0.44, 95% CI 0.19–1.21). Furthermore, the ganciclovir group results were moderately better than those obtained with the acyclovir group (OR = 0.38, 95% CI 0.12–0.71), while the valacyclovir group is superior to the acyclovir group (OR = 0.17, 95% CI: 0.03-0.72). The valganciclovir group showed no significant advantages than the acyclovir group in their efficacy and safety (OR = 0.31, 95% CI 0.05–1.04).

As CMV infection is one of the most important causes of rejection after transplantation, we have also analyzed the data of acute rejection after transplantation. For this analysis was included nine out of 17 studies with 1021 patients. However, our results did not show any significant statistical evidence (Additional file 1: Figure S2A). Besides this, we also tried to collect and analyze the data concerning the side effects caused by these drugs, such as leukopenia, Of the 17 articles, 10 studies comprising 1486 patients were eligible for the analysis. The results still show no significant difference (Additional file 1: Figure S2B).

### Subgroup analysis

Next, we conducted a subgroup analysis. In regard universal prophylaxis, the results were in line with the results described above. For the analysis of infection by CMV (Additional file 1: Figure S2C), 10 of the 17 studies were included [7, 14–22]. Both the ganciclovir and valacyclovir groups showed significant advantages compared with the control group (ganciclovir group: OR = 0.23, 95% CI 0.06–0.57; valacyclovir group: OR = 0.27, 95% CI 0.05–0.83). Of note, the valganciclovir group also showed significant statistical differences compared to the control group (OR = 0.22, 95% CI 0.03–0.79). The ganciclovir group showed better activity compared to the acyclovir group (OR = 0.36, 95% CI 0.11–0.77). However, the valacyclovir group showed no statistically significant difference compared with the acyclovir group (OR = 0.42, 95% CI 0.08–1.13).

As for CMV disease subgroup (Additional file 1: Figure S2D), 12 out of the 17 studies were included in the analysis [12, 23–26]. All three groups provided results were significantly better than the control group.(ganciclovir group: OR = 0.16, 95% CI 0.04–0.40; valacyclovir group: OR = 0.04, 95% CI 0.00–0.27; valganciclovir group: OR = 0.15, 95% CI 0.02–0.89), while the acyclovir group did not show significant differences (OR = 0.38, 95% CI 0.13–1.33). The results obtained with the ganciclovir group were superior to those obtained for the acyclovir group (OR = 0.41, 95% CI 0.10–0.97).

### Inconsistency assessment and prevention ranking

We conduct direct pairwise comparison for different outcomes to investigate the heterogeneity of the same intervention. The results of CMV infection (Additional file 1: Figure S3) showed that there is low-degree heterogeneity except when comparing valganciclovir and valacyclovir. About the direct pairwise comparisons of CMV disease (Additional file 1: Figure S4), there was detected only a moderate-degree heterogeneity between acyclovir and ganciclovir. As for acute rejection (Additional file 1: Figure S5A) and leukopenia (Additional file 1: Figure S5B), it was found a high degree of heterogeneity between valganciclovir and valacyclovir in acute rejection.

**Fig. 4:**
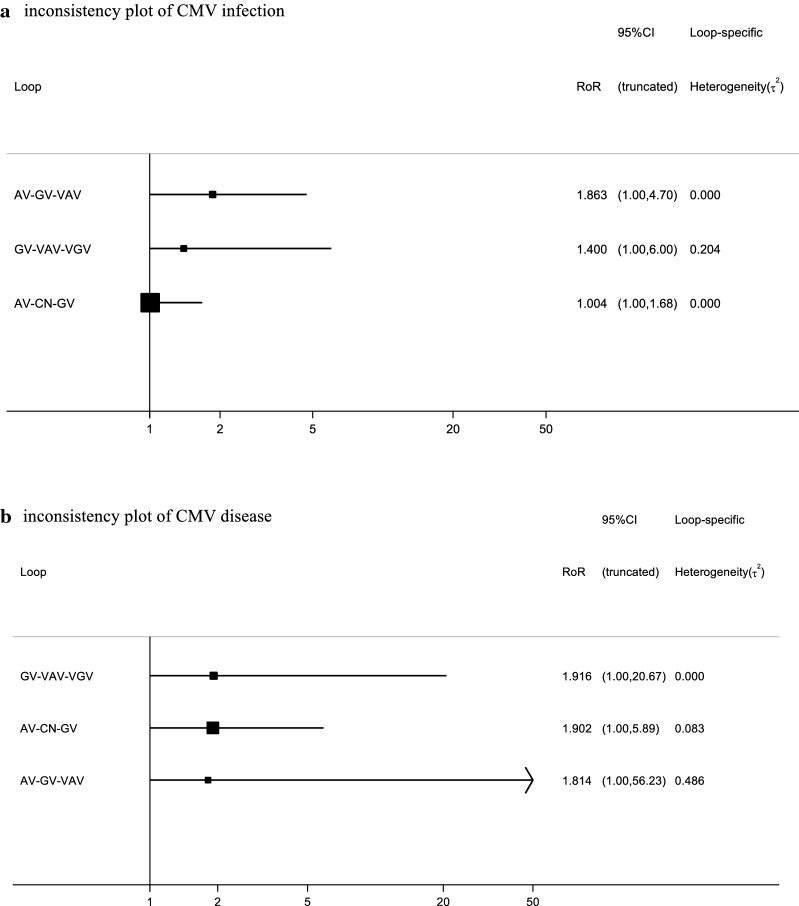
Inconsistency analysis for CMV infection (**a**) and CMV disease (**b**) in the network. The three independent closed loops are: ganciclovir-valacyclovir-valganciclovir, acyclovir-ganciclovir-valacyclovir and acyclovir- control-ganciclovir, respectively. The ROR value is close to one, indicating that the inconsistency is weak. *AV* acyclovir, *GV* ganciclovir, *VAV* valacyclovir, *VGV* valganciclovir, *CN* control, *CMV* cytomegalovirus

**Fig. 5:**
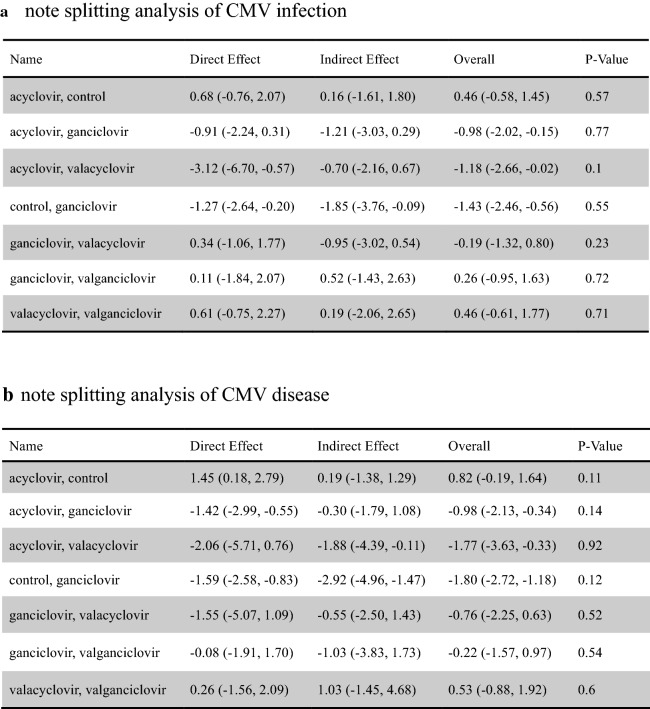
Node-splitting analysis for CMV infection (**a**) and CMV disease (**b**) in the network. All of the results compared direct and indirect evidence between different antiviral drugs did not show significant statistical differences (significant difference with p-values < 0.05). *CI* confidence interval, *CMV* cytomegalovirus

**Fig. 6:**
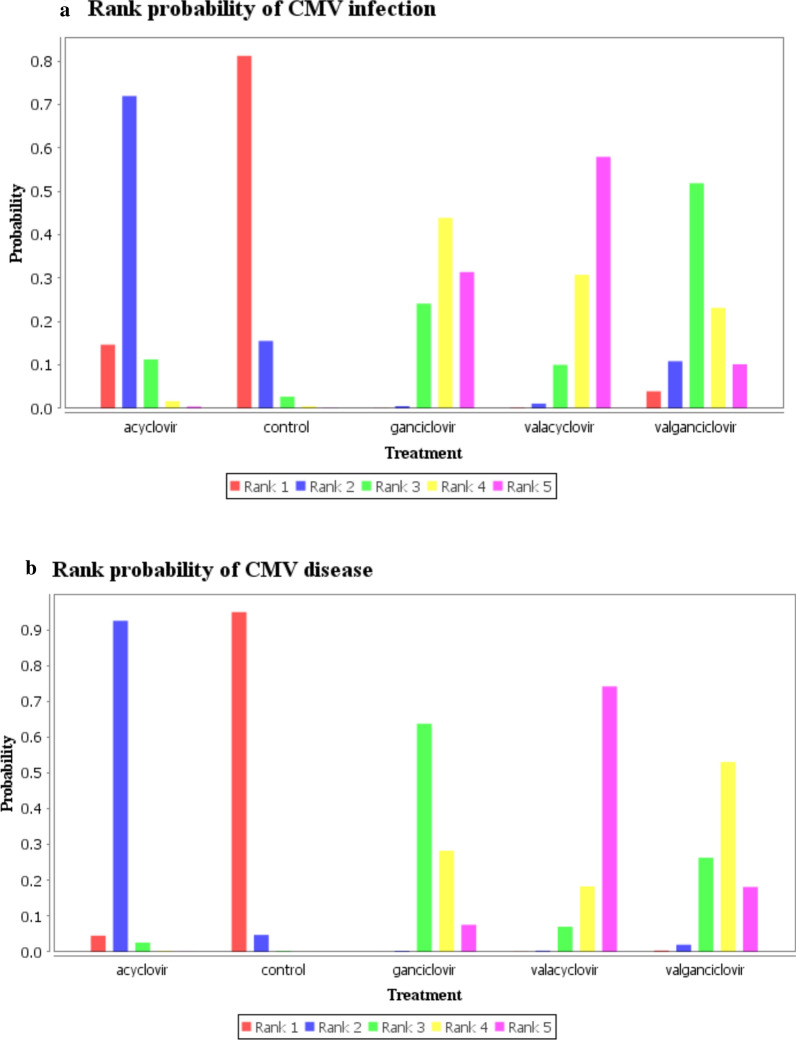
The ranking probabilities of CMV infection (**a**) and CMV disease (**b**). The figure shows the probability of each intervention being best, second best, third best, and so on. Rank 5 is the best because the less likely the occurrence of CMV infection and disease with the corresponding interventions

About the inconsistency analysis, as can be seen, there were three independent closed loops in the network, respectively, for CMV infection (Fig. 4a) and CMV disease (Fig. 4b): ganciclovir–valacyclovir–valganciclovir, acyclovir–ganciclovir–valacyclovir and acyclovir–control–ganciclovir. The ROR value obtained was close to one, indicating that the inconsistency is weak. We observed similar results in other outcome and subgroup analysis about inconsistency assessment (Additional file 1: Figure S6). Then node-splitting analyses were used to compare direct and indirect evidences between the different antiviral drugs. The results about CMV infection (Fig. 5a) and CMV disease (Fig. 5b) did not show significant statistical differences. The node-splitting analyses were used in other outcome and subgroup analyses also did not show significant statistical differences (Additional file 1: Figure S7). Therefore, we use the consistency model to analyze the data. According to the definition used, the lower incidence of the outcomes (CMV infection and CMV disease), the better effect of the antiviral drug. Based on the results of CMV infection (Fig. 6a), the most effective antiviral drugs may still be valacyclovir (0.59). The second and third are respectively ganciclovir (0.44) and valganciclovir (0.49), while the last one is acyclovir (0.71). As for CMV disease (Fig. 6b), valacyclovir (0.72) has the largest effect in preventing the occurrence of CMV disease after solid organ transplantation, followed by valganciclovir (0.53), ganciclovir (0.63) and acyclovir (0.92). In terms of acute rejection (Additional file 1: Figure S8A), the most probably induction order is acyclovir (0.57), ganciclovir (0.60), valganciclovir (0.47) and valacyclovir (0.63). Concerning the inducement of leukopenia (Additional file 1: Figure S8B), the safest antiviral drug was acyclovir (0.92) followed by ganciclovir (0.44), valacyclovir (0.46) and valganciclovir (0.56). In the subgroup analysis of universal prophylaxis, the most preferable prevention strategy for CMV disease (Additional file 1: Figure S8D) is valacyclovir (0.89), followed by valganciclovir (0.50), ganciclovir (0.54), acyclovir (0.86). It was not possible to assess what is the most effective drug for universal prophylaxis for CMV infection among these drugs (Additional file 1: Figure S8C).

### Publication bias

**Fig. 7:**
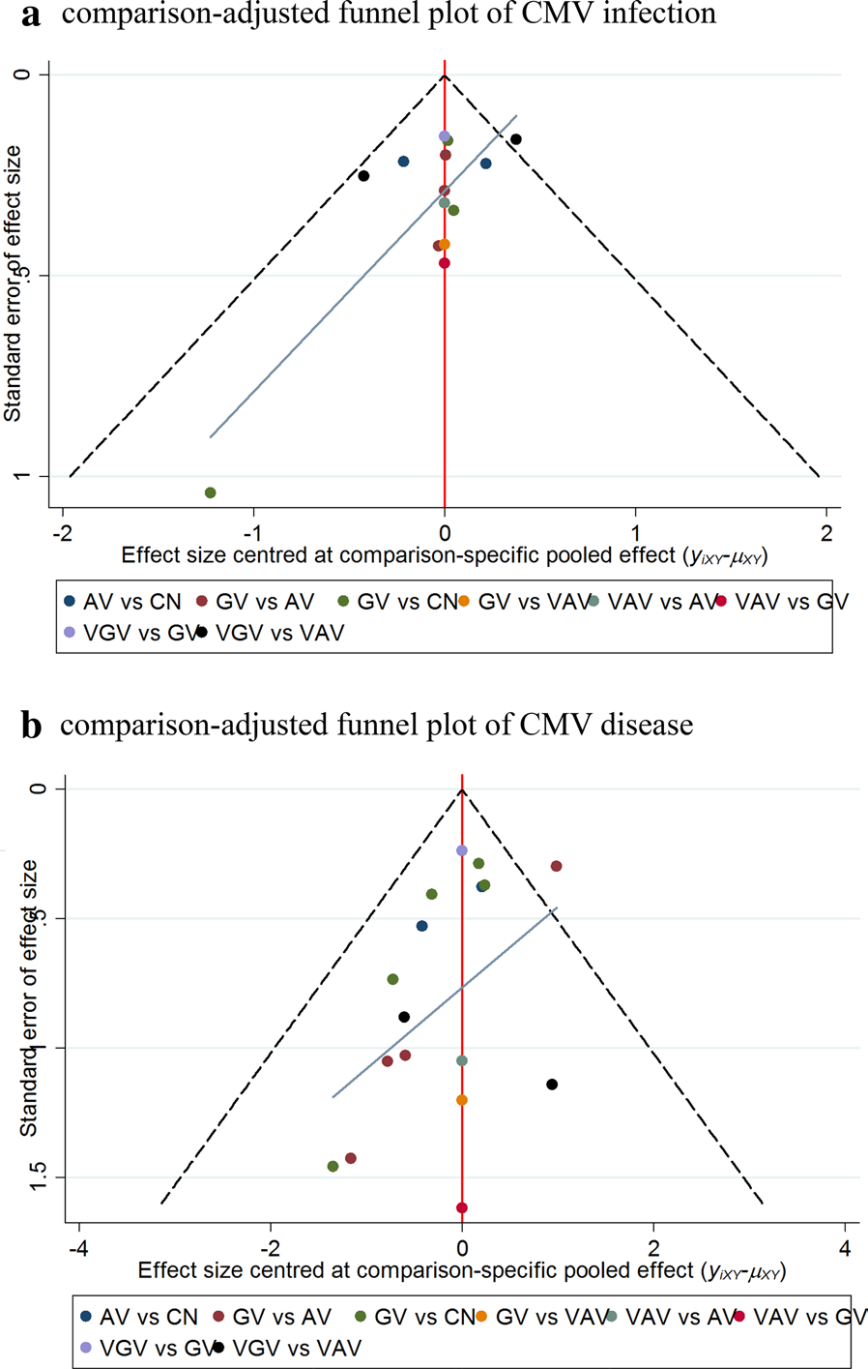
Comparison-adjusted funnel plots of CMV infection (**a**) and CMV disease (**b**). The red line suggests the null hypothesis that the study-specifc effect sizes do not differ from the respective comparison-specifc pooled effect estimates. The blue line is the regression line. Different colors represent different comparisons. *AV* acyclovir, *GV* ganciclovir, *VAV* valacyclovir, *VGV* valganciclovir, *CN* control, *CMV* cytomegalovirus

We evaluated the publication bias by comparison-adjusted funnel plots where the horizontal axis presents the difference between study specific size effect and the corresponding comparison specific summary effect. The funnel plot should be symmetrical near the zero line if there is no publication bias. The results showed no publication bias in regard to CMV infection studies (Fig. 7a), but small study effects were observed in CMV disease analysis (Fig. 7b). Therefore, we downgraded our confidence in the network and for the comparison ganciclovir versus valacyclovir about CMV disease after solid organ transplantation. We also analyzed the publication bias of other outcomes and subgroups (Additional file 1: Figure S9).

## Discussion

As far as we know, this is the first comprehensive network meta-analysis about the prevention of CMV after solid organ transplantation. Nowadays, there are several antiviral drugs for the treatment of CMV infection, such as acyclovir, valacyclovir, ganciclovir and valganciclovir. But there is lack of head-to-head clinical studies that compare between the available CMV antivirals, their activities and efficacy as well as the secondary effects they may cause. CMV infection and disease increase the risk of graft rejection and dysfunction after solid organ transplantation, which leads to increased morbidity and mortality. Nearly all antiviral drugs have side effects and can induce drug resistance after long-term use. Therefore in our network meta-analysis, we also focused on the side effects and resistance effects of the CMV antiviral drugs -acyclovir, valacyclovir, ganciclovir and valganciclovir. 17 studies involving 2062 patients were included in our network meta-analysis. From the results obtained from our network meta-analysis, valacyclovir and ganciclovir could effectively prevent CMV infection and disease after solid organ transplantation compared with the control group. Valganciclovir has also demonstrated a positive role in the prevention of CMV disease. Although the comparison between valganciclovir and control groups did not reach statistical significance. The valganciclovir group (CMV infection: p = 0.49) showed that it may have some advantages in the case of CMV infection after solid organ transplantation. The direct comparison between CMV infection and disease after solid organ transplantation, the preventive effect of valacyclovir and ganciclovir was significantly better than that of acyclovir. No significant differences between valacyclovir and ganciclovir were noted in prevention of CMV infection and disease after solid organ transplantation. We further wanted to explore the role of different antiviral drugs in different preventive measures (universal prophylaxis and preemptive therapy). Concerning preemptive therapy, due to the scarcity of studies we could not obtain enough data to perform an accurate analysis. For universal prophylaxis, the results of the network meta-analysis for the different antiviral drugs are similar to that obtained for the prevention of CMV infection and disease after solid organ transplantation. Compared with the control group, valacyclovir, valganciclovir and ganciclovir play an active role in the prevention of CMV infection and disease. Ganciclovir performed better than acyclovir in preventing CMV disease and infection, but valacyclovir was only better than acyclovir in the prevention of CMV disease. We compared acute rejection after transplantation and some indirect effects among the different antiviral drugs. Although the results of network meta-analysis did not show statistically significant differences between the different antiviral drugs, the trend of the incidence of acute rejection and indirect effects of the different antiviral drugs still had some clinical value. Acyclovir had the highest tendency for acute rejection, while valacyclovir was the lowest one. Acyclovir had the lowest probability to induce leukocytopenia and valganciclovir had the highest probability to induce.

Considering the positive effects and side effects, valacyclovir may have the largest effect on the prevention of CMV infection and disease, and the lowest risk of acute rejection after transplantation. However, the drawback of valacyclovir is that it may induce leukopenia. On the contrary, acyclovir may have the weakest therapeutic effect, with minimum chance of leukopenia occurrence. Compared to ganciclovir, valganciclovir is the most efficient in controlling CMV disease, but it is also the most likely to induce leukopenia among the four antiviral drugs. Nowadays, universal prophylaxis and preemptive therapy are the two mainly prevention strategies for the prevention of CMV infection and disease. In this sense, we performed a subgroup analysis. For the treatment of CMV disease, valacyclovir is still likely to be our best choice, followed by valganciclovir, ganciclovir and acyclovir. However, valacyclovir did not show significant advantages compared to valganciclovir and ganciclovir for the treatment of CMV infection.

There are several limitations in our research study. First, similar to other network analyses, we got the summary data from original studies rather than detailed data of every patient, it is not possible to analyse whether there is other prognostic factors that can affect the results obtained. Second, with increasing sensitivity to CMV infection and CMV disease diagnosis, there may be differences in the authenticity between the studies we included. Third, despite the lack of sufficient data available have limited our analysis for secondary effects, there was still some enough data to infer the probability of these antiviral drugs to induce leukopenia. The lack of sufficient data for analysis was the reason why we did not conduct subgroup analysis based on risk stratification.

In conclusion, in this study we compared the efficacy and adverse effects of different antiviral drugs for CMV treatment after solid organ transplantation. Our results may provide some clues for clinical treatment. Choosing antiviral drugs after transplantation needs comprehensive consideration, involving susceptibility, dose, economic factors, etc. Therefore, clinical selection of antiviral drugs should be individualized according to the current clinical presentation of the patients. Besides this, more comprehensive head-to-head clinical trials are needed to confirm the efficacy and side effects of the antiviral drugs for CMV treatment.

## Supplementary information

**Additional file 1: Figure S1.** Network plot of different outcome and subgroup analysis. Different nodes represent different treatments and the size of the nodes corresponds to the number of patients. The line represents a direct comparison between the two treatments and the thickness of the line is consistent with the number of direct comparisons of the two treatments. **Figure S2.** The results of Bayesian network meta-analysis. We should read result from right to left. Each result is a comparison between the column-defining treatment and the row-defining treatment. We highlight the data with significant statistical difference (p < 0.05) by *. (Abbreviations: CI, confidence interval; CMV, Cytomegalovirus.). **Figure S3.** Direct pairwise comparisons of CMV infection. There are five direct pairwise comparisons of antiviral drugs among the included studies. The heterogeneity was assessed by I2 statistic (low-degree:25-49%; moderate-degree:50–75%; highdegree: > 75%). There is a high-degree heterogeneity between the comparison between valganciclovir and valacyclovir. **Figure S4.** Direct pairwise comparisons of CMV disease. There are five direct pairwise comparisons of antiviral drugs among the included studies. The heterogeneity was assessed by I2 statistic (low-degree:25-49%; moderate-degree:50–75%; highdegree: > 75%). There is only a moderate-degree heterogeneity between the comparison between acyclovir and ganciclovir. **Figure S5.** Direct pairwise comparisons of acute rejection and leukopenia. There are two direct pairwise comparisons respectively among acute rejection and leukopenia. The heterogeneity was assessed by I2 statistic (low-degree:25-49%; moderate-degree:50–75%; high-degree: > 75%). About acute rejection, There is a low-degree heterogeneity between the comparison between ganciclovir and valacyclovir and a high-degree heterogeneity between the comparison between valganciclovir and valacyclovir. As for leukopenia, There is only a low-degree heterogeneity between the comparison between acyclovir and ganciclovir. **Figure S6.** Inconsistency analysis of different outcome and subgroup analysis in the network. The ROR value of all result is close to one, indicating that the inconsistency is weak. (Abbreviations: AV, acyclovir; GV, ganciclovir; VAV, valacyclovir; VGV, valganciclovir; CN, control.) **Figure S7.** Node-splitting analyses of different outcome and subgroup analysis in the network. All of the results compared direct and indirect evidence between different antiviral drugs did not show significant statistical differences (significant difference with p-values < 0.05). **Figure S8.** Rank possibility of different outcome and subgroup analysis. The figure shows the probability of each Intervention being best, second best, third best, and so on. Rank 5 is the best because the less likely the occurrence of CMV infection and disease with the corresponding interventions. **Figure S9.** Comparison-adjusted funnel plot of different outcome and subgroup analysis in the network. The red line suggests the null hypothesis that the study-specifc effect sizes do not differ from the respective comparison-specifc pooled effect estimates. The blue line is the regression line. Different colors represent different comparisons. The funnel plot should be symmetrical near the zero line if there is no publication bias (Abbreviations: AV, acyclovir; GV, ganciclovir; VAV, valacyclovir; VGV, valganciclovir; CN, control.).

## Data Availability

All relevant data are within the paper. If master chart required can be made available on request.
