# Supplementary material for: Prevention of cytomegalovirus infection after solid organ transplantation: a Bayesian network analysis
Source: Ann Clin Microbiol Antimicrob. 2020 Aug 5;19:34. doi: 10.1186/s12941-020-00372-0 (PMC7409489; doi:10.1186/s12941-020-00372-0)
Supplement: Supplementary file 1 — Additional file 1: Figure S1. Network plot of different outcome and subgroup analysis. Different nodes represent different treatments and the size of the nodes corresponds to the number of patients. The line represents a direct comparison between the two treatments and the thickness of the line is consistent with the number of direct comparisons of the two treatments. Figure S2. The results of Bayesian network meta-analysis. We should read result from right to left. Each result is a comparison between the column-defining treatment and the row-defining treatment. We highlight the data with significant statistical difference (p < 0.05) by *. (Abbreviations: CI, confidence interval; CMV, Cytomegalovirus.). Figure S3. Direct pairwise comparisons of CMV infection. There are five direct pairwise comparisons of antiviral drugs among the included studies. The heterogeneity was assessed by I2 statistic (low-degree:25-49%; moderate-degree:50–75%; highdegree: > 75%). There is a high-degree heterogeneity between the comparison between valganciclovir and valacyclovir. Figure S4. Direct pairwise comparisons of CMV disease. There are five direct pairwise comparisons of antiviral drugs among the included studies. The heterogeneity was assessed by I2 statistic (low-degree:25-49%; moderate-degree:50–75%; highdegree: > 75%). There is only a moderate-degree heterogeneity between the comparison between acyclovir and ganciclovir. Figure S5. Direct pairwise comparisons of acute rejection and leukopenia. There are two direct pairwise comparisons respectively among acute rejection and leukopenia. The heterogeneity was assessed by I2 statistic (low-degree:25-49%; moderate-degree:50–75%; high-degree: > 75%). About acute rejection, There is a low-degree heterogeneity between the comparison between ganciclovir and valacyclovir and a high-degree heterogeneity between the comparison between valganciclovir and valacyclovir. As for leukopenia, There is only a low-degree heterogeneity between the co [file 12941_2020_372_MOESM1_ESM.pdf]

# Additional file 1: Figure S1

Network plot of disease

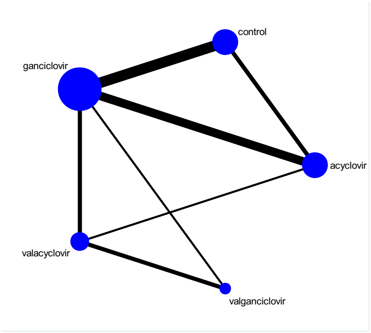


Network plot of infection

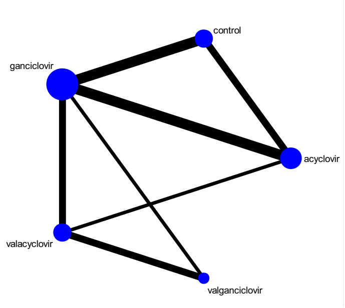


Network plot of acute rejection

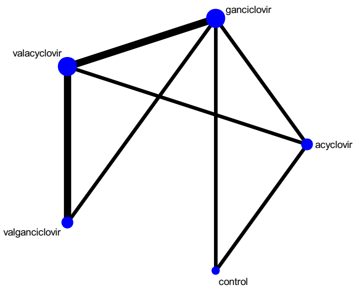


Network plot of acute rejection

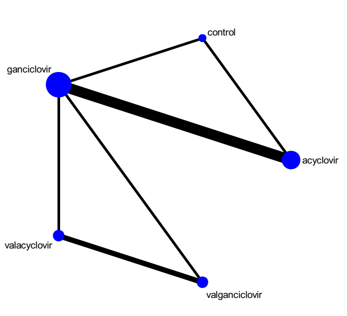


Network plot of subgroup disease

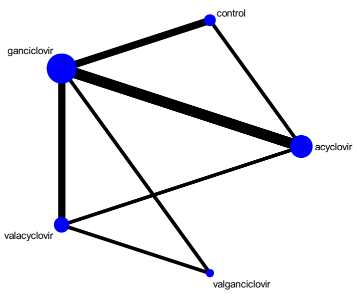


Network plot of subgroup infection

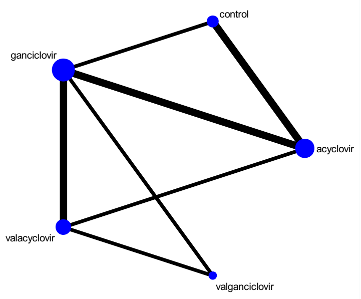

## Additional file 1: Figure S2

A: Odds ratios (OR) with 95% CI for acute rejection

| | | | | |
|---|---|---|---|---|
| acyclovir | 1.43 (0.21, 11.00) | 0.46 (0.08, 2.07) | 0.23 (0.03, 1.35) | 0.31 (0.03, 2.11) |
| | control | 0.32 (0.02, 2.86) | 0.16 (0.01, 1.68) | 0.22 (0.01, 2.57) |
| | | ganciclovir | 0.51 (0.14, 1.58) | 0.68 (0.17, 2.52) |
| | | | valacyclovir | 1.34 (0.43, 4.47) |
| | | | | valganciclovir |

B: Odds ratios (OR) with 95% CI for leukopenia

| | | | | |
|---|---|---|---|---|
| acyclovir | 3.73 (0.78, 25.71) | 2.61 (0.77, 8.83) | 3.54 (0.55, 25.45) | 5.02 (0.70, 29.94) |
| | control | 0.73 (0.11, 2.98) | 0.96 (0.08, 7.54) | 1.41 (0.11, 9.29) |
| | | ganciclovir | 1.30 (0.31, 6.21) | 1.82 (0.42, 7.38) |
| | | | valacyclovir | 1.48 (0.32, 5.11) |
| | | | | valganciclovir |

C: Odds ratios (OR) with 95% CI for CMV infection of subgroup analysis

| | | | | |
|---|---|---|---|---|
| acyclovir | 1.58 (0.58, 3.86) | 0.37 (0.12, 0.78)* | 0.43 (0.09, 1.13) | 0.35 (0.06, 1.07) |
| | control | 0.23 (0.07, 0.55)* | 0.28 (0.05, 0.81)* | 0.23 (0.04, 0.78)* |
| | | ganciclovir | 1.17 (0.41, 2.74) | 0.95 (0.28, 2.66) |
| | | | valacyclovir | 0.81 (0.26, 2.58) |
| | | | | valganciclovir |

D: Odds ratios (OR) with 95% CI for CMV disease of subgroup analysis

| | | | | |
|---|---|---|---|---|
| acyclovir | 2.67 (0.74, 7.74) | 0.44 (0.11, 0.95)* | 0.12 (0.01, 0.57)* | 0.42 (0.04, 1.83) |
| | control | 0.16 (0.04, 0.40)* | 0.05 (0.00, 0.26)* | 0.15 (0.02, 0.83)* |
| | | ganciclovir | 0.29 (0.04, 1.50) | 0.94 (0.20, 4.39) |
| | | | valacyclovir | 3.25 (0.51, 29.87) |
| | | | | valganciclovir |

# Additional file 1: Figure S3

## Direct pairwise comparisons of infection

| Study or Subgroup | Acyclovir Events | Acyclovir Total | Control Events | Control Total | Weight | Odds Ratio M-H, Fixed, 95% CI |
|---|---|---|---|---|---|---|
| Balfour, H. H., Jr. 1989 | 19 | 53 | 31 | 51 | 73.2% | 0.36 [0.16, 0.80] |
| Barkholt, L. 1999 | 16 | 28 | 17 | 27 | 26.8% | 0.78 [0.27, 2.31] |
| **Total (95% CI)** | | **81** | | **78** | **100.0%** | **0.47 [0.25, 0.89]** |
| Total events | 35 | | 48 | | | |

Heterogeneity: $Chi^2 = 1.29$, df = 1 (P = 0.26); $I^2 = 22\%$
Test for overall effect: Z = 2.30 (P = 0.02)

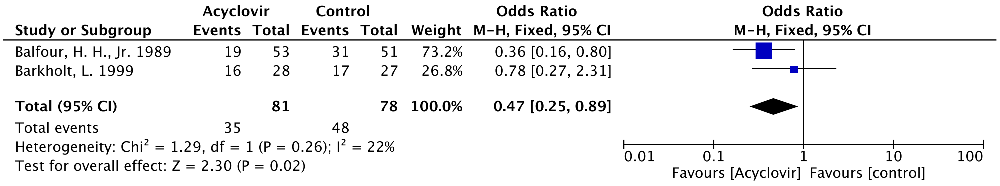


| Study or Subgroup | acyclovir Events | acyclovir Total | ganciclovir Events | ganciclovir Total | Weight | Odds Ratio M-H, Fixed, 95% CI | Year |
|---|---|---|---|---|---|---|---|
| Duncan, S. R. 1994 | 10 | 12 | 7 | 13 | 6.5% | 4.29 [0.66, 27.78] | 1994 |
| Singh, N. 1994 | 10 | 24 | 6 | 23 | 20.7% | 2.02 [0.59, 6.96] | 1994 |
| Rubin, R. H. 2000 | 39 | 78 | 25 | 77 | 72.8% | 2.08 [1.08, 3.99] | 2000 |
| **Total (95% CI)** | | **114** | | **113** | **100.0%** | **2.21 [1.28, 3.83]** | |
| Total events | 59 | | 38 | | | | |

Heterogeneity: $Chi^2 = 0.54$, df = 2 (P = 0.77); $I^2 = 0\%$
Test for overall effect: Z = 2.83 (P = 0.005)

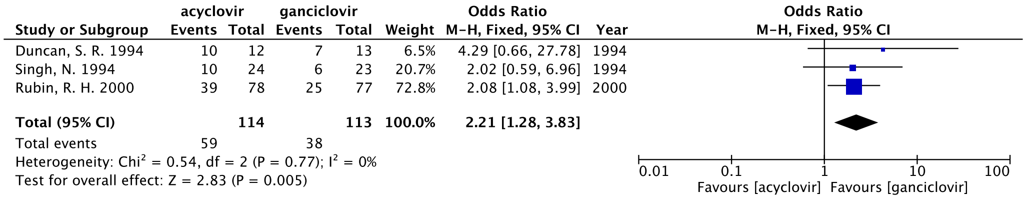


| Study or Subgroup | ganciclovir Events | ganciclovir Total | Control Events | Control Total | Weight | Odds Ratio M-H, Fixed, 95% CI | Year |
|---|---|---|---|---|---|---|---|
| Hibberd, P. L. 1995 | 11 | 64 | 17 | 64 | 17.7% | 0.57 [0.24, 1.35] | 1995 |
| Gane, E. 1997 | 37 | 150 | 79 | 154 | 73.7% | 0.31 [0.19, 0.51] | 1997 |
| Paya, C. V. 2002 | 1 | 35 | 7 | 34 | 8.7% | 0.11 [0.01, 0.98] | 2002 |
| **Total (95% CI)** | | **249** | | **252** | **100.0%** | **0.34 [0.23, 0.51]** | |
| Total events | 49 | | 103 | | | | |

Heterogeneity: $Chi^2 = 2.57$, df = 2 (P = 0.28); $I^2 = 22\%$
Test for overall effect: Z = 5.13 (P < 0.00001)

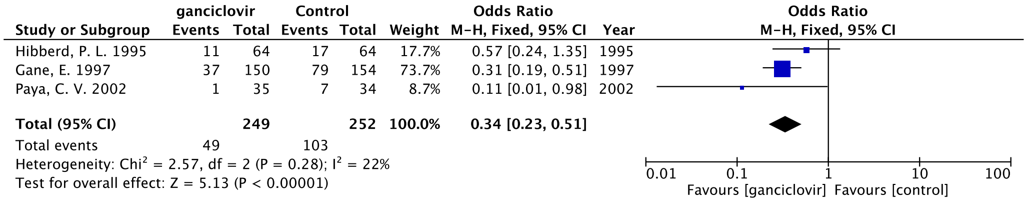


| Study or Subgroup | ganciclovir Events | ganciclovir Total | valacyclovir Events | valacyclovir Total | Weight | Odds Ratio M-H, Fixed, 95% CI | Year |
|---|---|---|---|---|---|---|---|
| Pavlopoulou, I. D. 2005 | 7 | 43 | 8 | 43 | 42.7% | 0.85 [0.28, 2.60] | 2005 |
| Reischig, T. 2005 | 7 | 36 | 11 | 35 | 57.3% | 0.53 [0.18, 1.57] | 2005 |
| **Total (95% CI)** | | **79** | | **78** | **100.0%** | **0.67 [0.31, 1.45]** | |
| Total events | 14 | | 19 | | | | |

Heterogeneity: $Chi^2 = 0.36$, df = 1 (P = 0.55); $I^2 = 0\%$
Test for overall effect: Z = 1.03 (P = 0.30)

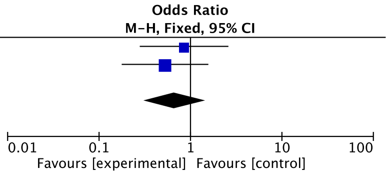


| Study or Subgroup | valganciclovir Events | valganciclovir Total | valacyclovir Events | valacyclovir Total | Weight | Odds Ratio M-H, Fixed, 95% CI | Year |
|---|---|---|---|---|---|---|---|
| Reischig, T. 2012 | 33 | 36 | 19 | 34 | 8.8% | 8.68 [2.22, 33.90] | 2012 |
| Reischig, T. 2015 | 18 | 60 | 24 | 59 | 91.2% | 0.63 [0.29, 1.33] | 2015 |
| **Total (95% CI)** | | **96** | | **93** | **100.0%** | **1.33 [0.73, 2.43]** | |
| Total events | 51 | | 43 | | | | |

Heterogeneity: $Chi^2 = 11.11$, df = 1 (P = 0.0009); $I^2 = 91\%$
Test for overall effect: Z = 0.94 (P = 0.35)

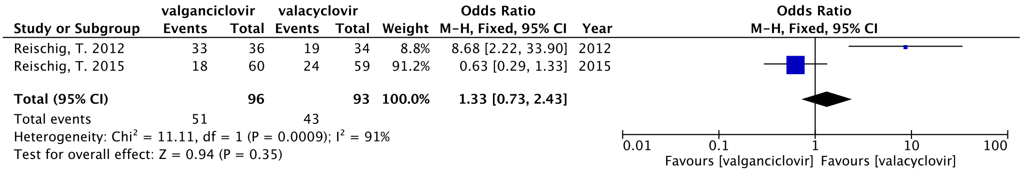

## Additional file 1: Figure S4

# Direct pairwise comparisons of CMV disease

| Study or Subgroup | Acyclovir Events | Acyclovir Total | Ganciclovir Events | Ganciclovir Total | Weight | Odds Ratio M-H, Fixed, 95% CI |
|---|---|---|---|---|---|---|
| Duncan, S. R. 1994 | 7 | 24 | 1 | 23 | 5.6% | 9.06 [1.02, 80.84] |
| Rubin, R. H. 2000 | 5 | 12 | 0 | 13 | 2.1% | 19.80 [0.96, 409.62] |
| Singh, N. 1994 | 21 | 78 | 15 | 77 | 85.2% | 1.52 [0.72, 3.24] |
| Winston, D. J. 2003 | 8 | 109 | 1 | 110 | 7.1% | 8.63 [1.06, 70.25] |
| **Total (95% CI)** | | **223** | | **223** | **100.0%** | **2.84 [1.54, 5.26]** |
| Total events | 41 | | 17 | | | |

Heterogeneity: $Chi^2 = 6.36, df = 3 (P = 0.10); I^2 = 53\%$
Test for overall effect: Z = 3.33 (P = 0.0009)

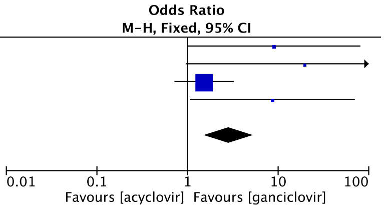


| Study or Subgroup | Acyclovir Events | Acyclovir Total | Control Events | Control Total | Weight | Odds Ratio M-H, Fixed, 95% CI |
|---|---|---|---|---|---|---|
| Balfour, H. H., Jr. 1989 | 4 | 53 | 15 | 51 | 56.9% | 0.20 [0.06, 0.64] |
| Barkholt, L. 1999 | 7 | 28 | 14 | 27 | 43.1% | 0.31 [0.10, 0.97] |
| **Total (95% CI)** | | **81** | | **78** | **100.0%** | **0.24 [0.11, 0.55]** |
| Total events | 11 | | 29 | | | |

Heterogeneity: $Chi^2 = 0.30, df = 1 (P = 0.58); I^2 = 0\%$
Test for overall effect: Z = 3.38 (P = 0.0007)

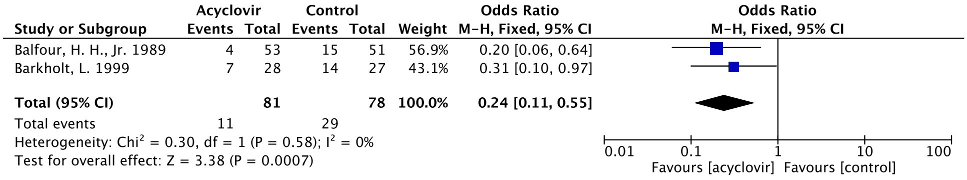


| Study or Subgroup | Ganciclovir Events | Ganciclovir Total | Control Events | Control Total | Weight | Odds Ratio M-H, Fixed, 95% CI |
|---|---|---|---|---|---|---|
| Gane, E. 1997 | 7 | 150 | 29 | 154 | 31.9% | 0.21 [0.09, 0.50] |
| Hibberd, P. L. 1995 | 9 | 64 | 16 | 49 | 18.2% | 0.34 [0.13, 0.85] |
| Merigan, T. C. 1992 | 13 | 76 | 31 | 73 | 30.6% | 0.28 [0.13, 0.60] |
| Paya, C. V. 2002 | 0 | 35 | 5 | 34 | 6.4% | 0.08 [0.00, 1.42] |
| Sagedal, S. 2003 | 2 | 42 | 11 | 38 | 12.9% | 0.12 [0.03, 0.60] |
| **Total (95% CI)** | | **367** | | **348** | **100.0%** | **0.23 [0.15, 0.37]** |
| Total events | 31 | | 92 | | | |

Heterogeneity: $Chi^2 = 2.07, df = 4 (P = 0.72); I^2 = 0\%$
Test for overall effect: Z = 6.28 (P < 0.00001)

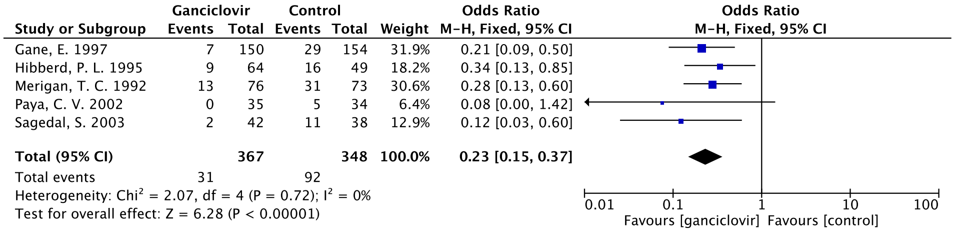


| Study or Subgroup | ganciclovir Events | ganciclovir Total | valacyclovir Events | valacyclovir Total | Weight | Odds Ratio M-H, Fixed, 95% CI | Year |
|---|---|---|---|---|---|---|---|
| Pavlopoulou, I. D. 2005 | 1 | 40 | 0 | 43 | 32.7% | 3.30 [0.13, 83.47] | 2005 |
| Reischig, T. 2005 | 2 | 36 | 1 | 35 | 67.3% | 2.00 [0.17, 23.11] | 2005 |
| **Total (95% CI)** | | **76** | | **78** | **100.0%** | **2.43 [0.35, 16.88]** | |
| Total events | 3 | | 1 | | | | |

Heterogeneity: $Chi^2 = 0.06, df = 1 (P = 0.81); I^2 = 0\%$
Test for overall effect: Z = 0.90 (P = 0.37)

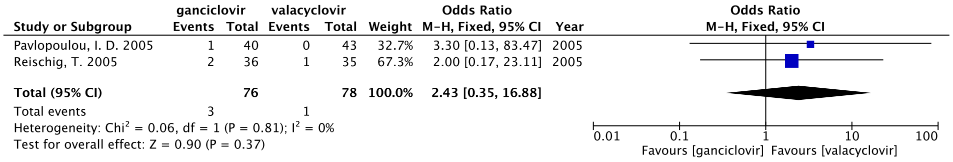


| Study or Subgroup | Valganciclovir Events | Valganciclovir Total | Valacyclovir Events | Valacyclovir Total | Weight | Odds Ratio M-H, Fixed, 95% CI | Year |
|---|---|---|---|---|---|---|---|
| Reischig, T. 2012 | 2 | 36 | 3 | 34 | 75.3% | 0.61 [0.10, 3.88] | 2012 |
| Reischig, T. 2015 | 3 | 60 | 1 | 59 | 24.7% | 3.05 [0.31, 30.22] | 2015 |
| **Total (95% CI)** | | **96** | | **93** | **100.0%** | **1.21 [0.32, 4.66]** | |
| Total events | 5 | | 4 | | | | |

Heterogeneity: $Chi^2 = 1.16, df = 1 (P = 0.28); I^2 = 13\%$
Test for overall effect: Z = 0.28 (P = 0.78)

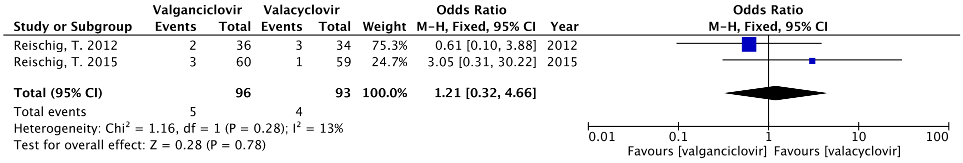

# Additional file 1: Figure S5

## A: direct pairwise comparisons of acute rejection

| Study or Subgroup | ganciclovir Events | ganciclovir Total | valacyclovir Events | valacyclovir Total | Weight | Odds Ratio M-H, Fixed, 95% CI | Year |
|---|---|---|---|---|---|---|---|
| Pavlopoulou, I. D. 2005 | 5 | 40 | 5 | 43 | 60.9% | 1.09 [0.29, 4.07] | 2005 |
| Reischig, T. 2005 | 12 | 36 | 4 | 35 | 39.1% | 3.88 [1.11, 13.54] | 2005 |
| **Total (95% CI)** | | **76** | | **78** | **100.0%** | **2.18 [0.90, 5.23]** | |
| Total events | 17 | | 9 | | | | |

Heterogeneity: Chi² = 1.88, df = 1 (P = 0.17); I² = 47%
Test for overall effect: Z = 1.74 (P = 0.08)

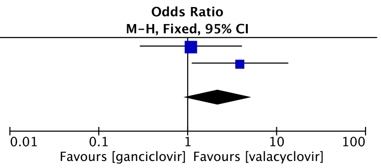


| Study or Subgroup | valganciclovir Events | valganciclovir Total | valacyclovir Events | valacyclovir Total | Weight | Odds Ratio M-H, Fixed, 95% CI |
|---|---|---|---|---|---|---|
| Reischig, T. 2012 | 15 | 36 | 6 | 34 | 19.2% | 3.33 [1.11, 10.04] |
| Reischig, T. 2015 | 10 | 60 | 18 | 59 | 80.8% | 0.46 [0.19, 1.09] |
| **Total (95% CI)** | | **96** | | **93** | **100.0%** | **1.01 [0.53, 1.91]** |
| Total events | 25 | | 24 | | | |

Heterogeneity: Chi² = 7.67, df = 1 (P = 0.006); I² = 87%
Test for overall effect: Z = 0.03 (P = 0.98)

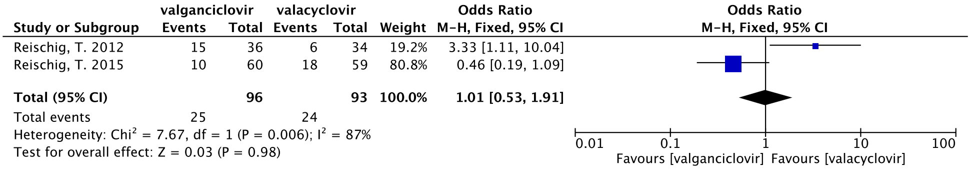


## B: direct pairwise comparisons of leukopenia

| Study or Subgroup | acyclovir Events | acyclovir Total | ganciclovir Events | ganciclovir Total | Weight | Odds Ratio M-H, Fixed, 95% CI | Year |
|---|---|---|---|---|---|---|---|
| Duncan, S. R. 1994 | 0 | 12 | 0 | 13 | | Not estimable | 1994 |
| Singh, N. 1994 | 1 | 24 | 0 | 23 | 1.1% | 3.00 [0.12, 77.47] | 1994 |
| Rubin, R. H. 2000 | 2 | 78 | 12 | 77 | 27.3% | 0.14 [0.03, 0.66] | 2000 |
| Winston, D. J. 2003 | 20 | 109 | 38 | 110 | 71.6% | 0.43 [0.23, 0.79] | 2003 |
| **Total (95% CI)** | | **223** | | **223** | **100.0%** | **0.38 [0.22, 0.65]** | |
| Total events | 23 | | 50 | | | | |

Heterogeneity: Chi² = 3.26, df = 2 (P = 0.20); I² = 39%
Test for overall effect: Z = 3.47 (P = 0.0005)

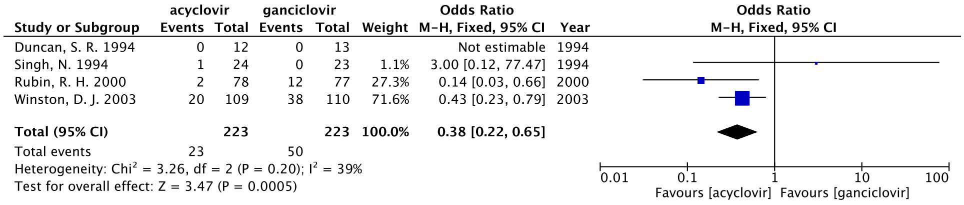


| Study or Subgroup | valganciclovir Events | valganciclovir Total | valacyclovir Events | valacyclovir Total | Weight | Odds Ratio M-H, Fixed, 95% CI | Year |
|---|---|---|---|---|---|---|---|
| Reischig, T. 2012 | 0 | 36 | 1 | 34 | 14.8% | 0.31 [0.01, 7.77] | 2012 |
| Reischig, T. 2015 | 20 | 60 | 13 | 59 | 85.2% | 1.77 [0.78, 4.00] | 2015 |
| **Total (95% CI)** | | **96** | | **93** | **100.0%** | **1.55 [0.71, 3.37]** | |
| Total events | 20 | | 14 | | | | |

Heterogeneity: Chi² = 1.07, df = 1 (P = 0.30); I² = 6%
Test for overall effect: Z = 1.11 (P = 0.27)

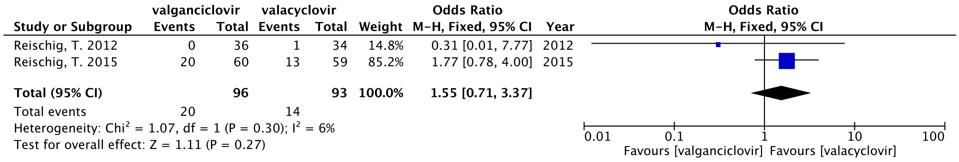

# Additional file 1: Figure S6

A: inconsistency plot of acute rejection

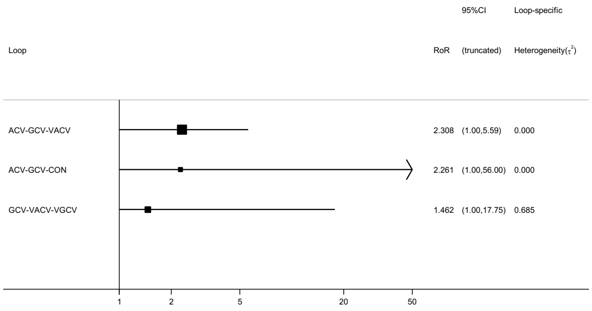


B: inconsistency plot of leukopenia

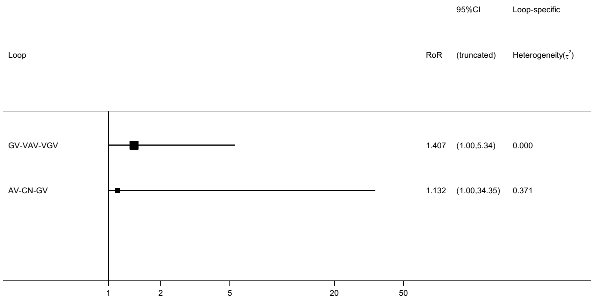


C: inconsistency plot of subgroup CMV infection

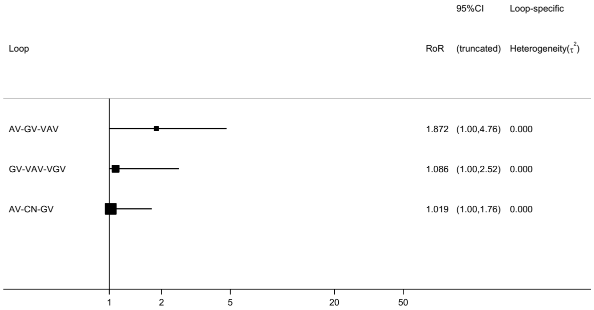


D: inconsistency plot of subgroup CMV disease

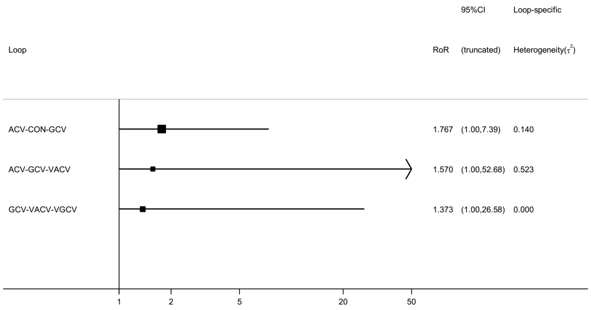

# Additional file 1: Figure S7

A: note splitting analysis of acute rejection

| Name | Direct Effect | Indirect Effect | Overall | P-Value |
|---|---|---|---|---|
| acyclovir, control | 0.16 (-1.89, 2.30) | 7.17 (-1.62, 27.66) | 0.36 (-1.54, 2.40) | 0.19 |
| acyclovir, ganciclovir | -0.48 (-2.29, 1.39) | -12.14 (-51.31, -0.12) | -0.77 (-2.56, 0.73) | 0.08 |
| acyclovir, valacyclovir | -10.62 (-36.11, 0.62) | -1.27 (-3.49, 0.78) | -1.47 (-3.63, 0.30) | 0.2 |
| control, ganciclovir | -7.04 (-25.65, 1.28) | -0.72 (-3.45, 1.83) | -1.13 (-3.80, 1.05) | 0.2 |
| ganciclovir, valacyclovir | -0.80 (-2.33, 0.79) | -0.54 (-2.90, 1.55) | -0.68 (-1.96, 0.46) | 0.82 |
| ganciclovir, valganciclovir | -0.18 (-2.04, 1.66) | -0.72 (-2.78, 1.34) | -0.39 (-1.77, 0.92) | 0.67 |
| valacyclovir, valganciclovir | 0.13 (-1.28, 1.61) | 0.70 (-1.65, 3.12) | 0.29 (-0.85, 1.50) | 0.63 |

B: note splitting analysis of leukopenia

| Name | Direct Effect | Indirect Effect | Overall | P-Value |
|---|---|---|---|---|
| acyclovir, control | 1.41 (-1.51, 4.99) | 1.55 (-1.18, 4.11) | 1.32 (-0.25, 3.25) | 0.94 |
| acyclovir, ganciclovir | 1.03 (-0.52, 2.39) | 0.94 (-2.56, 5.08) | 0.96 (-0.26, 2.18) | 0.97 |
| control, ganciclovir | -0.57 (-2.65, 1.53) | -0.36 (-5.33, 2.66) | -0.31 (-2.22, 1.09) | 0.93 |
| ganciclovir, valacyclovir | 0.09 (-2.32, 2.32) | 0.54 (-1.95, 3.88) | 0.26 (-1.16, 1.83) | 0.73 |
| ganciclovir, valganciclovir | 0.83 (-1.32, 3.05) | 0.31 (-3.13, 2.94) | 0.60 (-0.86, 2.00) | 0.72 |
| valacyclovir, valganciclovir | 0.21 (-2.05, 1.82) | 0.78 (-2.29, 4.14) | 0.39 (-1.15, 1.63) | 0.71 |

C: note splitting analysis of subgroup CMV infection

| Name | Direct Effect | Indirect Effect | Overall | P-Value |
|---|---|---|---|---|
| acyclovir, control | 0.75 (-0.66, 2.02) | -0.08 (-2.59, 1.84) | 0.46 (-0.54, 1.35) | 0.35 |
| acyclovir, ganciclovir | -0.94 (-2.59, 0.42) | -1.29 (-3.51, 0.21) | -0.99 (-2.16, -0.25) | 0.72 |
| acyclovir, valacyclovir | -3.36 (-6.63, -0.92) | -0.25 (-1.44, 0.77) | -0.85 (-2.41, 0.12) | 0.03 |
| control, ganciclovir | -1.19 (-2.84, 0.58) | -1.93 (-4.00, -0.38) | -1.45 (-2.66, -0.59) | 0.34 |
| ganciclovir, valacyclovir | 0.36 (-1.04, 1.82) | -0.18 (-2.69, 1.27) | 0.15 (-0.90, 1.01) | 0.57 |
| ganciclovir, valganciclovir | 0.11 (-1.72, 2.03) | -0.53 (-2.90, 1.67) | -0.06 (-1.28, 0.98) | 0.52 |
| valacyclovir, valganciclovir | -0.47 (-2.43, 1.47) | 0.16 (-2.02, 2.58) | -0.21 (-1.34, 0.95) | 0.53 |

D: note splitting analysis of subgroup CMV disease

| Name | Direct Effect | Indirect Effect | Overall | P-Value |
|---|---|---|---|---|
| acyclovir, control | 1.39 (0.03, 2.96) | 0.35 (-2.04, 1.93) | 0.98 (-0.30, 2.05) | 0.2 |
| acyclovir, ganciclovir | -1.33 (-3.50, -0.24) | -0.12 (-2.02, 1.81) | -0.83 (-2.25, -0.06) | 0.19 |
| acyclovir, valacyclovir | -2.24 (-5.92, 0.87) | -2.45 (-5.66, -0.13) | -2.14 (-4.34, -0.56) | 0.96 |
| control, ganciclovir | -1.45 (-2.96, -0.16) | -2.78 (-5.06, -1.11) | -1.81 (-3.15, -0.91) | 0.18 |
| ganciclovir, valacyclovir | -1.50 (-5.48, 1.27) | -1.25 (-3.92, 1.36) | -1.25 (-3.23, 0.41) | 0.92 |
| ganciclovir, valganciclovir | -0.11 (-2.37, 2.11) | 0.29 (-3.74, 4.58) | -0.06 (-1.60, 1.48) | 0.83 |
| valacyclovir, valganciclovir | 1.40 (-1.72, 5.14) | 1.26 (-1.80, 4.47) | 1.18 (-0.68, 3.40) | 0.92 |

# Additional file 1: Figure S8

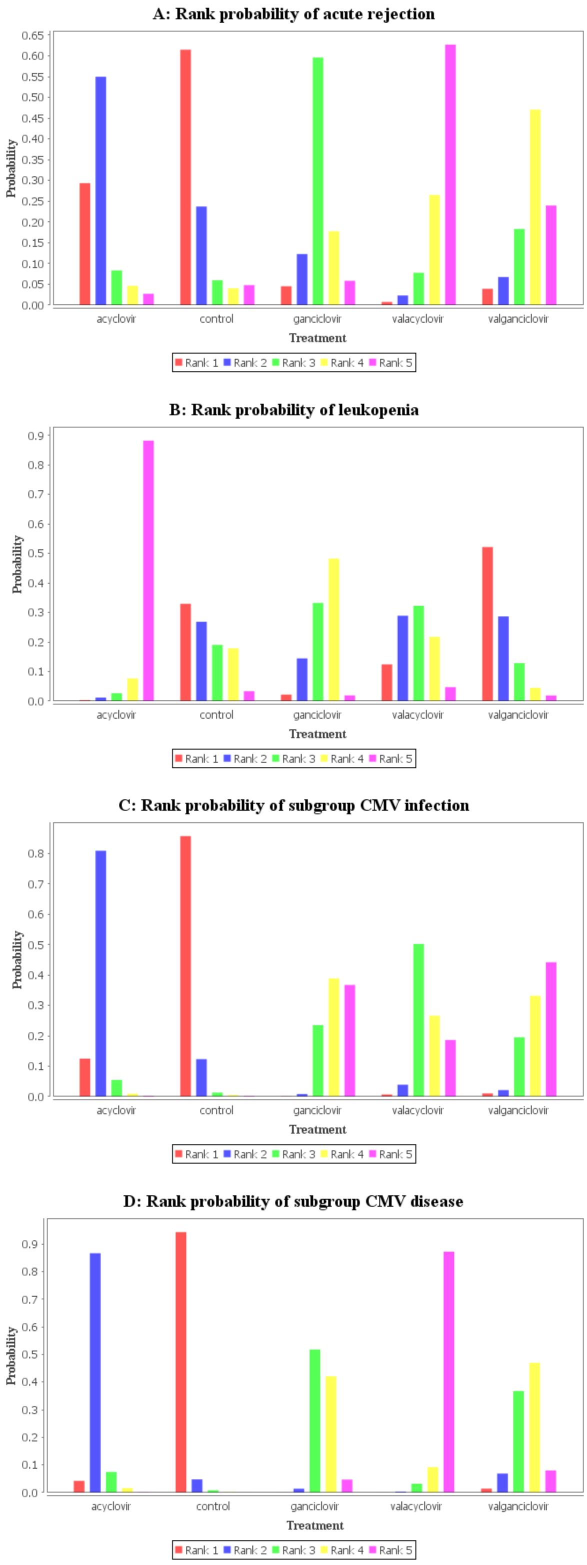

Additional file 1: Figure S9

A: comparison-adjusted funnel plot of acute rejection

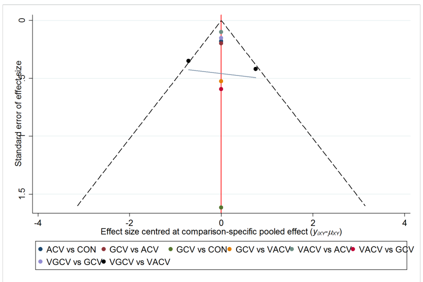


B: comparison-adjusted funnel plot of leukopenia

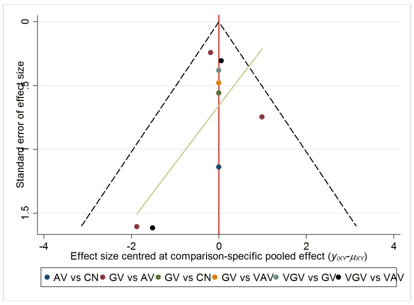


C: comparison-adjusted funnel plot of subgroup CMV infection

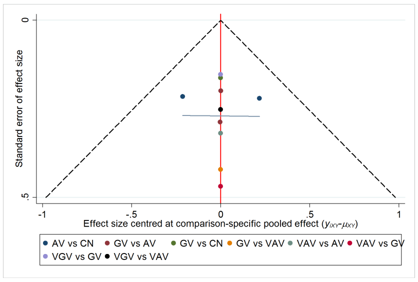


C: comparison-adjusted funnel plot of subgroup CMV disease

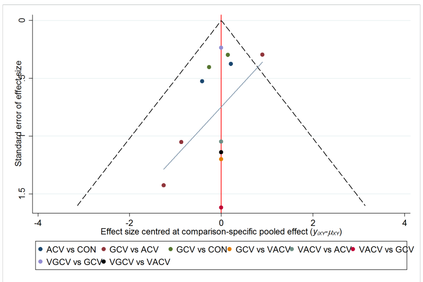


## Supplementary material figure legends

Figure S1 Network plot of different outcome and subgroup analysis. Different nodes represent different treatments and the size of the nodes corresponds to the number of patients. The line represents a direct comparison between the two treatments and the thickness of the line is consistent with the number of direct comparisons of the two treatments.

Figure S2 The results of Bayesian network meta-analysis. We should read result from right to left. Each result is a comparison between the column-defining treatment and the row-defining treatment. We highlight the data with significant statistical difference

($p < 0.05$) by *. (Abbreviations: CI, confidence interval; CMV, Cytomegalovirus.)

Figure S3 Direct pairwise comparisons of CMV infection. There are five direct pairwise comparisons of antiviral drugs among the included studies. The heterogeneity was assessed by $I^2$ statistic (low-degree:25-49%; moderate-degree:50–75%; high-degree: >75%). There is a high-degree heterogeneity between the comparison between valganciclovir and valacyclovir.

Figure S4 Direct pairwise comparisons of CMV disease. There are five direct pairwise comparisons of antiviral drugs among the included studies. The heterogeneity was assessed by $I^2$ statistic (low-degree:25-49%; moderate-degree:50–75%; high-degree: >75%). There is only a moderate-degree heterogeneity between the comparison between acyclovir and ganciclovir.

Figure S5 Direct pairwise comparisons of acute rejection and leukopenia. There are two direct pairwise comparisons respectively among acute rejection and leukopenia. The heterogeneity was assessed by $I^2$ statistic (low-degree:25-49%; moderate-degree:50–75 %; high-degree: >75%). About acute rejection, There is a low-degree heterogeneity between the comparison between ganciclovir and valacyclovir and a high-degree

heterogeneity between the comparison between valganciclovir and valacyclovir. As for leukopenia, There is only a low-degree heterogeneity between the comparison between acyclovir and ganciclovir.

Figure S6 Inconsistency analysis of different outcome and subgroup analysis in the network. The ROR value of all result is close to one, indicating that the inconsistency is weak. ( Abbreviations: AV, acyclovir; GV, ganciclovir; VAV, valacyclovir; VGV, valganciclovir; CN, control.)

Figure S7 Node-splitting analyses of different outcome and subgroup analysis in the network. All of the results compared direct and indirect evidence between different antiviral drugs did not show significant statistical differences (significant difference with p-values <0.05).

Figure S8 Rank possibility of different outcome and subgroup analysis. The figure shows the probability of each Intervention being best, second best, third best, and so on . Rank 5 is the best because the less likely the occurrence of CMV infection and disease with the corresponding interventions

Figure S9 Comparison-adjusted funnel plot of different outcome and subgroup analysis

in the network. The red line suggests the null hypothesis that the study-specifc effect sizes do not differ from the respective comparison-specifc pooled effect estimates. The blue line is the regression line. Different colors represent different comparisons. The funnel plot should be symmetrical near the zero line if there is no publication bias (Abbreviations: AV, acyclovir; GV, ganciclovir; VAV, valacyclovir; VGV, valganciclovir; CN, control.)
